# Supplementary figures and images for: Analysis and Simulation of Glioblastoma Cell Lines-Derived Extracellular Vesicles Metabolome
Source: Metabolites. 2020 Mar 2;10(3):88. doi: 10.3390/metabo10030088 (PMC7142482; doi:10.3390/metabo10030088)

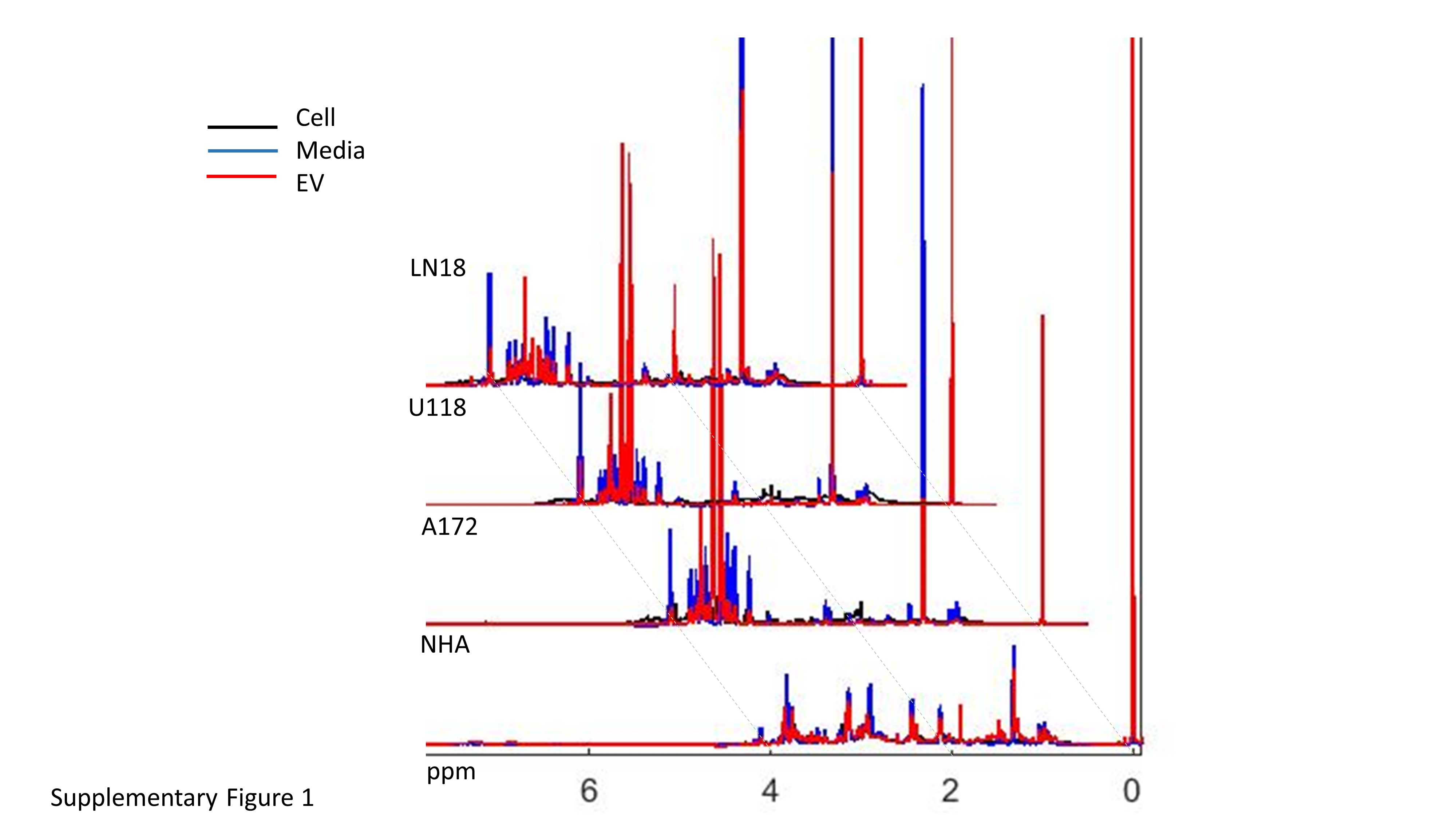

Supplement: Supplementary file 1 [file metabolites-10-00088-s001.zip › supplementary_figure1.jpg]

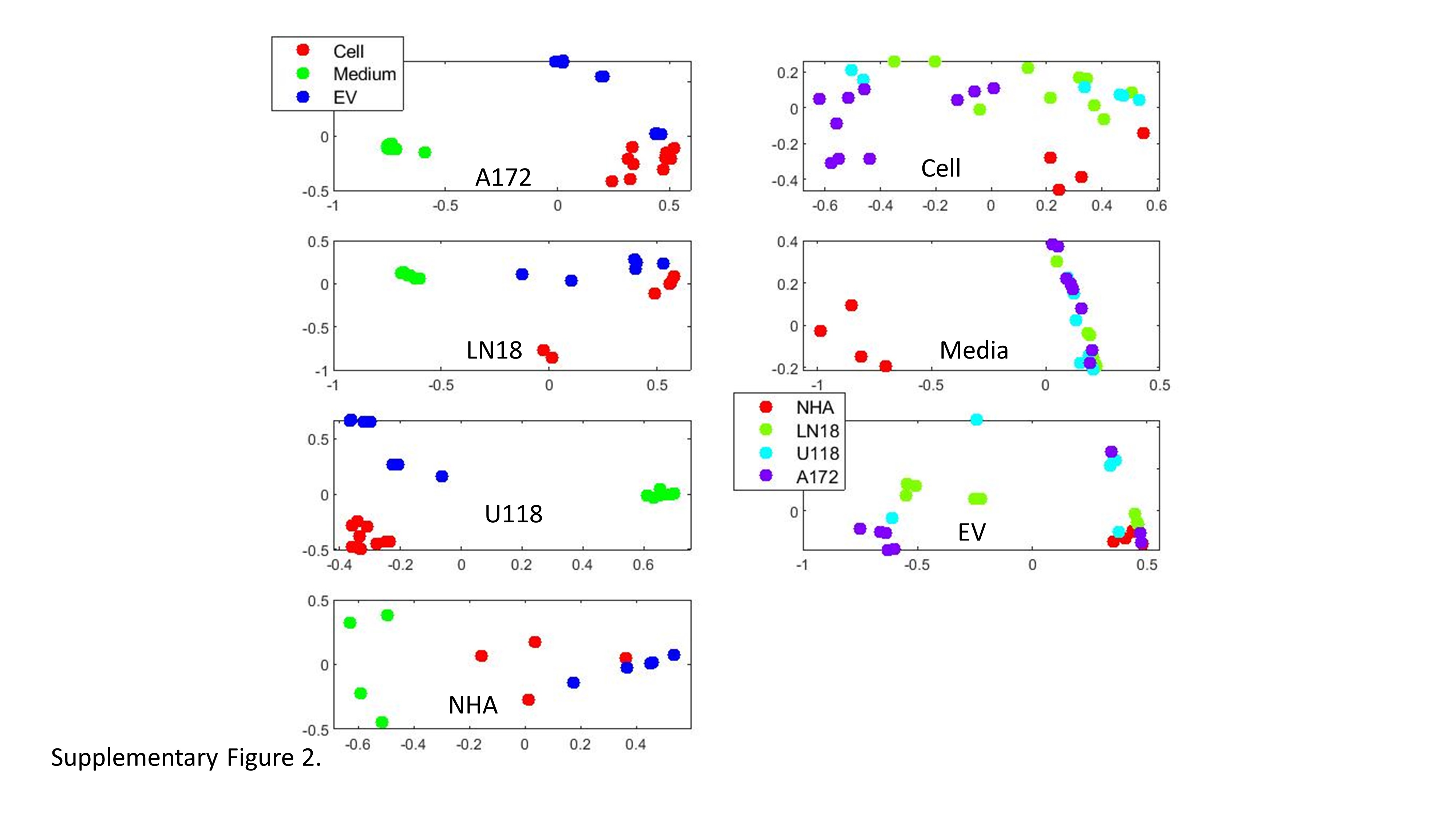

Supplement: Supplementary file 1 [file metabolites-10-00088-s001.zip › supplementary_figure2.jpg]

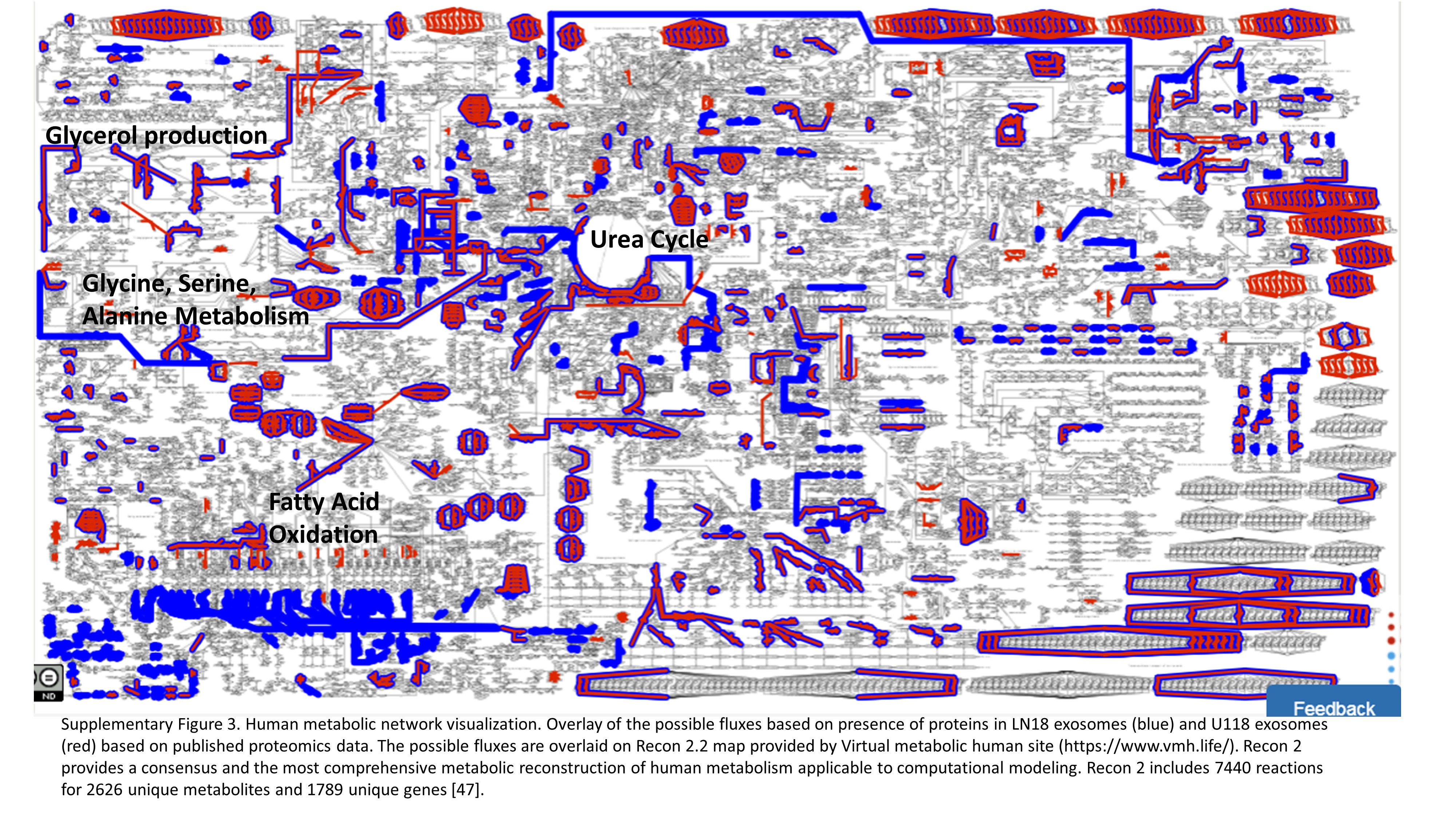

Supplement: Supplementary file 1 [file metabolites-10-00088-s001.zip › supplementary_figure3.jpg]
